# Ruxolitinib-Associated Improvement in Post-transplant Air-Leak Syndrome Complicating Steroid-Dependent Organizing Pneumonia: A Case Report

**DOI:** 10.7759/cureus.112618

**Published:** 2026-07-13

**Authors:** Takahiro Maeta, Kentaro Kobayashi, Kazuya Asano, Tsuyoshi Sato, Shigeki Ito

**Affiliations:** 1 Hematology and Oncology, Iwate Medical University, Yahaba, JPN

**Keywords:** air-leak syndrome, chronic graft-versus-host disease, cryptogenic organizing pneumonia, hematopoietic stem cell transplantation, pneumomediastinum, ruxolitinib

## Abstract

Air-leak syndrome (ALS), including pneumothorax, pneumomediastinum, and subcutaneous emphysema, is a rare but life-threatening complication after allogeneic hematopoietic stem cell transplantation (HSCT). However, no standard treatment has yet been established. Here, we report the case of a 60-year-old man with acute myeloid leukemia who underwent allogeneic HSCT. He developed chronic graft-versus-host disease (GVHD) and cryptogenic organizing pneumonia (COP) on day 126 after transplantation. Although initial corticosteroid therapy improved the COP, the relapse occurred during tapering, and repeated steroid treatment failed to achieve sustained disease control. The patient subsequently developed pneumomediastinum, consistent with ALS. Ruxolitinib treatment was initiated on day 265 for steroid-dependent chronic GVHD and organizing pneumonia. Following treatment, oxygenation improved, pneumomediastinum improved, and corticosteroids were successfully tapered and discontinued without COP recurrence. Serum Krebs von den Lungen-6 (KL-6) levels decreased in parallel with clinical and radiological improvement.

The introduction of ruxolitinib is associated with improvements in ALS and enabled the discontinuation of steroids. Adverse events included cytopenia and mild liver dysfunction, consistent with known safety profiles. The patient died of acute respiratory failure after transfusion, which was not considered a direct complication of the ruxolitinib treatment.

This case suggests that ruxolitinib may facilitate corticosteroid tapering and radiographic improvement of organizing pneumonia-associated ALS after allogeneic transplantation.

## Introduction

Air-leak syndrome (ALS), which includes pneumothorax, pneumomediastinum (PM), also referred to as mediastinal emphysema, and subcutaneous emphysema, is a rare complication associated with allogeneic hematopoietic stem cell transplantation (HSCT) [1,2]. Patients with ALS have significantly worse survival outcomes than those without ALS [1-3]. ALS is diagnosed based on chest radiography and/or computed tomography (CT) findings. Pneumothorax is defined as the presence of extra-alveolar air in one or both hemithoraces, PM as air in the mediastinal space, and subcutaneous emphysema as air in subcutaneous tissues [1]. Pleural drainage is an option for patients with pneumothorax; however, no standard treatment for ALS has been established [1]. ALS has been reported to develop following chronic graft-versus-host disease (GVHD) and cryptogenic organizing pneumonia (COP) [1,3]. Bronchiolitis obliterans syndrome (BOS), a pulmonary manifestation of chronic GVHD, is thought to result from fibrosis of the peripheral airways caused by recurrent inflammatory lesions triggered by activated CD8-positive T cells, aberrant expression of MHC class II antigens, and the expression of minor histocompatibility antigens in lung tissue, ultimately leading to decreased lung compliance [1,4]. Polypoid intraluminal occlusion is characterized by the proliferation of granulation tissue consisting of fibroblasts, myofibroblasts, and loose connective tissue in COP [5]. Alveolar rupture can result from increased intra-alveolar pressure, alveolar wall damage, or both, leading to the development of ALS [1]. Chronic GVHD is often observed in patients after HSCT, leading to non-relapse mortality and an impaired quality of life [6]. Systemic glucocorticoids are the first-line treatment for chronic GVHD; however, approximately 50% of the patients become glucocorticoid-refractory or glucocorticoid-dependent [7]. Additionally, COP is a complication associated with allogeneic HSCT and leads to non-relapse mortality [8,9].

Ruxolitinib is an orally administered, selective inhibitor of Janus kinase (JAK)1/2 [10]. JAKs are intracellular tyrosine kinases that play a critical role in immune-cell development and function and are involved in the pathogenesis of GVHD [10]. In the REACH3 study, patients treated with ruxolitinib showed better responses, longer failure-free survival, and manageable safety profiles than those in the control group [7]. Combination therapy with PSL and ruxolitinib has been reported to be effective for glucocorticoid-refractory COP [5]. Although ruxolitinib has been reported to be effective in chronic GVHD and COP, its efficacy in patients with ALS has not been reported. Because ruxolitinib has shown efficacy in chronic GVHD and COP, which are underlying conditions associated with ALS, it may also contribute to the improvement of ALS through steroid-sparing effects.

Here, we report a case of ALS after allogeneic HSCT in which pneumomediastinum improved following the initiation of ruxolitinib.

## Case presentation

**Figure 1:**
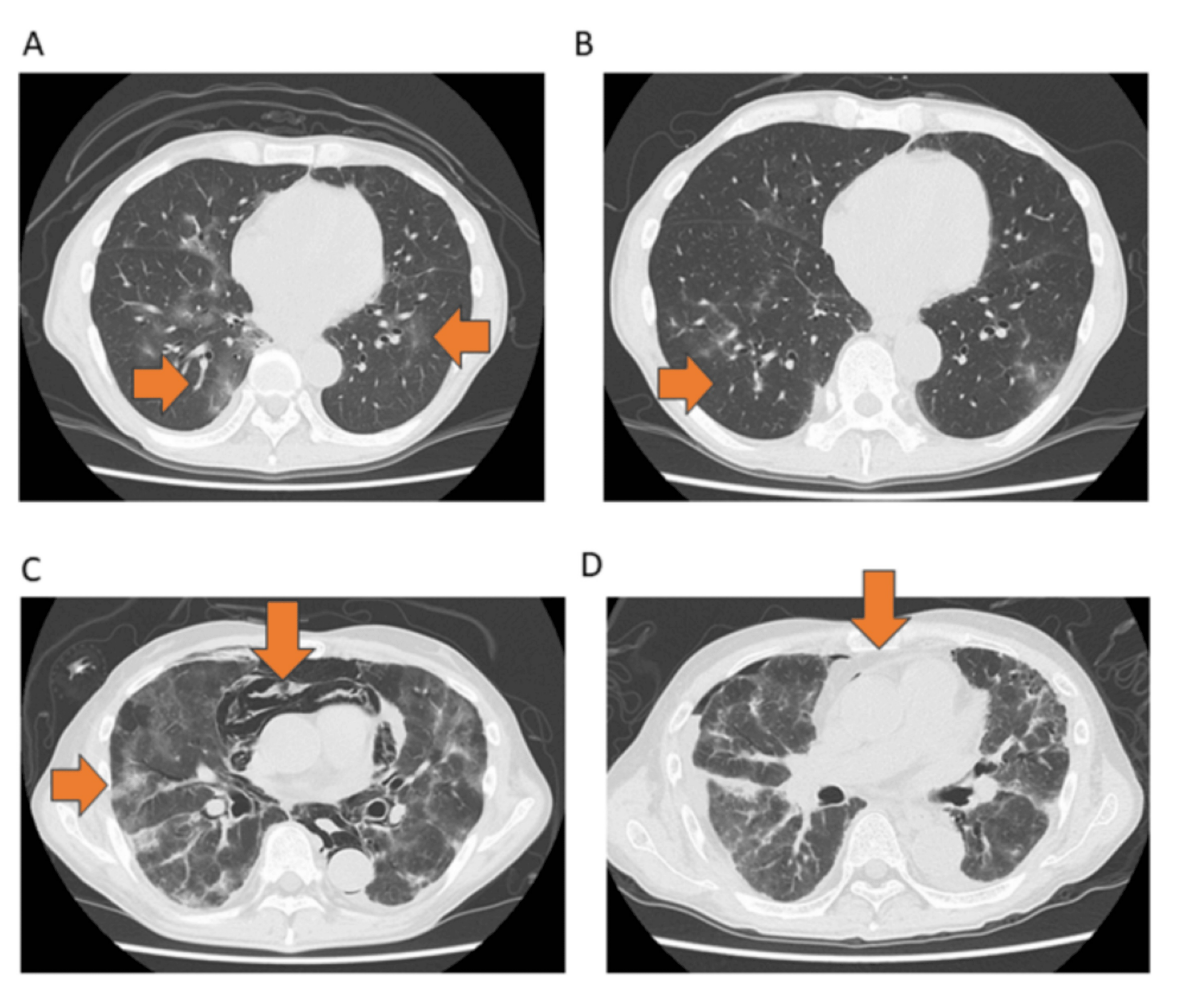
CT images of the patient. (A) Chest CT performed on day 126 after PBSCT revealed bilateral diffuse ground-glass opacities with infiltrative shadows. The arrows indicate the areas of diffuse ground-glass opacities with infiltrative shadows. (B) Chest CT performed on day 150 after PBSCT showed improvement in COP. The arrow indicates the resolution of the ground-glass opacities and infiltrative shadows. (C) Chest CT performed on day 261 after PBSCT revealed bilateral diffuse ground-glass opacities with infiltrative shadows and confirmed the presence of pneumomediastinum. The arrows indicate the areas of diffuse ground-glass opacities with infiltrative shadows and pneumomediastinum. (D) Chest CT performed on day 342 after PBSCT, corresponding to 77 days after the initiation of ruxolitinib treatment, showed improvement in pneumomediastinum. The arrow indicates the resolution of the pneumomediastinum. CT, computed tomography; PBSCT, peripheral blood stem cell transplantation; COP, cryptogenic organizing pneumonia

**Figure 2:**
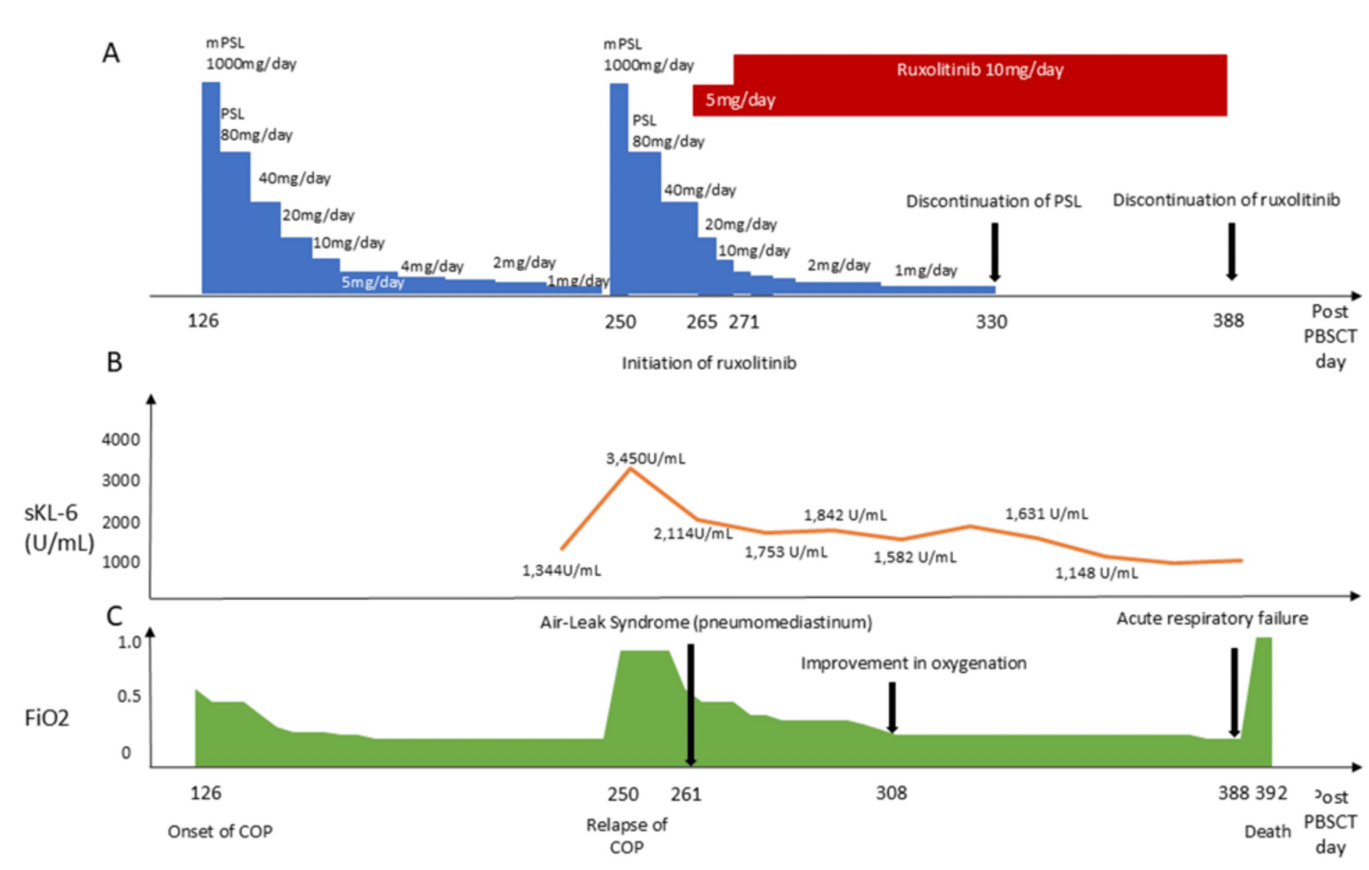
Clinical course (A) Details of medication treatment: Methylprednisolone pulse therapy (1 g/day for three days) was administered. Following methylprednisolone pulse therapy, the patient received PSL at 80 mg/day for one week, followed by 40 mg/day for one week, 20 mg/day for one week, and 10 mg/day for one week. Then, the dose was tapered by 5 mg weekly to 5 mg/day and subsequently by 1 mg every two weeks. PSL was discontinued on day 249 after PBSCT. Steroid pulse therapy with methylprednisolone 1 g/day was administered for three days from day 250 after PBSCT. The patient subsequently received PSL at 80 mg/day for one week, followed by 40 mg/day for one week. Ruxolitinib was initiated at 5 mg/day on day 265 after PBSCT, and then the ruxolitinib dose was increased from 5 to 10 mg/day on day 271 after PBSCT. Following the initiation of ruxolitinib therapy, the PSL dose was gradually tapered as follows: 20 mg/day for three days, 10 mg/day for three days, 5 mg/day for five days, 4 mg/day for five days, 3 mg/day for five days, 2 mg/day for 21 days, and 1 mg/day for 21 days. (B) Trends in serum KL-6 levels. (C) Trends in oxygen demand. mPSL, methylprednisolone; sKL-6, serum Krebs von den Lungen-6; PBSCT, peripheral blood stem cell transplantation; PSL, prednisolone; FiO₂, fraction of inspired oxygen; COP, cryptogenic organizing pneumonia

A 60-year-old Japanese man was diagnosed with acute myeloid leukemia in May 2021 and received induction therapy with idarubicin plus cytarabine (Ara-C), mitoxantrone plus Ara-C, and daunorubicin plus Ara-C. However, bone marrow hematopoiesis failed to recover, and hematologic relapse occurred during follow-up. The patient was subsequently treated with azacitidine and venetoclax, and a morphologically leukemia-free state was observed. Following a myeloablative conditioning regimen consisting of fludarabine (125 mg/m^2^) and busulfan (12.8 mg/kg), the patient underwent peripheral blood stem cell transplantation (PBSCT) from a human leukocyte antigen-matched donor in November 2022. As prophylaxis for acute GVHD, cyclosporine was initiated on day -1, and short-course methotrexate was administered. He developed lichen planus-like skin, depigmentation, dry eyes, and arthritis. Therefore, he was clinically diagnosed with chronic GVHD without biopsy [11], and cyclosporine adjustments were made. On day 126 after PBSCT, the patient developed a cough and dyspnea. Chest CT revealed bilateral diffuse ground-glass opacities (GGOs) with infiltrative shadows (Figure 1A). Spirometry revealed a percent vital capacity (%VC) of 77.0% and a forced expiratory volume in one second/forced vital capacity ratio (FEV1.0%) of 89.8%, consistent with restrictive ventilatory impairment. Bronchoalveolar lavage fluid analysis showed a CD4/CD8 ratio of 0.5, and bacterial cultures and polymerase chain reaction (PCR) assays for cytomegalovirus (CMV), human herpesvirus 6 (HHV-6), varicella-zoster virus (VZV), Pneumocystis jirovecii, Candida, and Aspergillus were all negative. The patient was diagnosed with COP. Methylprednisolone pulse therapy (1 g/day for three days) was administered, and oxygen therapy was initiated (Figures 2A, 2C). Following methylprednisolone pulse therapy, the patient received prednisolone (PSL) at 80 mg/day for one week, followed by 40 mg/day for one week, 20 mg/day for one week, and 10 mg/day for one week. Oxygen therapy was subsequently discontinued (Figure 2C). Thereafter, the dose was tapered by 5 mg weekly to 5 mg/day and subsequently by 1 mg every two weeks. The patient’s radiographic findings had improved by day 150 after PBSCT (Figure 1B). Steroid therapy was discontinued after four months; however, hypoxemia recurred the following day. CT revealed a combination of infiltrative shadows and diffuse bilateral GGOs, and infectious etiologies were considered less likely based on negative results for nasopharyngeal respiratory virus panel PCR, CMV-antigenemia, blood culture tests, and serum galactomannan antigen. Therefore, the patient was clinically diagnosed with relapsed COP on day 250 after PBSCT. Pulmonary function testing and BAL were not performed at the time of COP relapse because of severe dyspnea and respiratory instability. The serum Krebs von den Lungen-6 (KL-6) level was 3,450 U/mL at the time of COP relapse (Figure 2B). As in the previous episode, steroid pulse therapy with methylprednisolone 1 g/day was administered for three days from day 250 after PBSCT. The patient subsequently received PSL at 80 mg/day for one week, followed by 40 mg/day for one week. However, chest pain developed, and chest CT confirmed PM without pneumothorax or subcutaneous emphysema on day 261 after PBSCT (Figure 1C). Despite the administration of high-dose corticosteroid therapy, the serum KL-6 level remained persistently elevated at 2,114 U/mL on day 264 after PBSCT. Ruxolitinib 5 mg/day was initiated on day 265 after PBSCT because of glucocorticoid-refractory chronic GVHD [7], and ruxolitinib 5 mg/day was continued for six days. The ruxolitinib dose was increased from 5 to 10 mg/day on day 271 after PBSCT. Following the initiation of ruxolitinib therapy, the PSL dose was gradually tapered as follows: 20 mg/day for three days, 10 mg/day for three days, 5 mg/day for five days, 4 mg/day for five days, 3 mg/day for five days, 2 mg/day for twenty-one days, and 1 mg/day for twenty-one days. At the onset of ALS, the patient required high-flow nasal cannula oxygen therapy with a flow rate of 45 L/min and an FiO₂ of 0.40; however, by day 308 after PBSCT, 43 days after ruxolitinib initiation, the oxygen requirement had improved to nasal cannula oxygen therapy at 2 L/min, corresponding to an estimated FiO₂ of 0.24 (Figures 2A, 2C). PSL was discontinued on day 330 after PBSCT, corresponding to 65 days after the initiation of ruxolitinib, because of improved disease control. The serum KL-6 level was 1,148 U/mL, and chest CT showed an improvement in PM (Figure 1D) on day 342 after PBSCT, corresponding to 77 days after the initiation of ruxolitinib. Laboratory data obtained before the initiation of ruxolitinib were as follows: hemoglobin level, 11.4 g/dL; platelet count, 33,000/μL; neutrophil count, 1,320/μL; and alanine aminotransferase, 30 U/L. The nadir hemoglobin level and platelet count were 7.1 g/dL and 18,000/μL, respectively, three weeks after the initiation of ruxolitinib. The patient became transfusion-dependent and required weekly transfusions. The neutrophil count remained persistently at approximately 1,000/µL, while the alanine aminotransferase level increased to 140 U/L. Adverse events associated with ruxolitinib included anemia (Common Terminology Criteria for Adverse Events (CTCAE) version 5.0, grade 2), thrombocytopenia (grade 4), neutropenia (grade 2), and increased alanine aminotransferase level (grade 2). No infectious complications occurred during ruxolitinib treatment. No dose reduction or interruption of ruxolitinib was performed after dose escalation to 10 mg/day. On day 388 after PBSCT, the patient developed sudden respiratory failure and cardiac arrest immediately after the transfusion therapy. CT revealed bilateral pulmonary edema and cardiomegaly. Transfusion-related acute lung injury was clinically suspected; however, transfusion-associated circulatory overload and cardiogenic pulmonary edema could not be completely excluded. Ruxolitinib was discontinued because oral administration was no longer possible on day 388 after PBSCT. The patient died of respiratory failure on day 392 after PBSCT (Figure 2). Radiographic improvement of ALS was assessed based on chest CT findings [1]. Adverse events were evaluated using CTCAE, version 5.0 [12].

## Discussion

Non-infectious pulmonary complications, including BOS, COP, and ALS, are post-transplant complications that can be fatal [1,6,7,9]. The incidence rate of ALS has been reported to be 1.2% in Japan [1], and the prognosis has been reported to be poor [2]. No standard treatment has yet been established [1]. In this report, the initiation of ruxolitinib was associated with improved oxygenation, a decrease in KL-6 levels, radiographic improvement of PM, and successful corticosteroid reduction and discontinuation without COP relapse. However, because this is a single case report, a causal relationship between ruxolitinib and the improvement of ALS cannot be established.

ALS is caused by alveolar rupture [1]. Alveolar rupture may result from increased intra-alveolar pressure, damage to the alveolar walls, or a combination of both factors [1]. In addition, systemic steroid therapy has been shown to negatively affect wound healing [13]. In this case, we speculated that the ALS resulted from the progression of fibrosis associated with COP, leading to alveolar rupture. Additionally, systemic steroid therapy may impair alveolar tissue repair, further contributing to the development of ALS. Although PM may have spontaneously improved, the patient received repeated steroid pulse therapy before the initiation of ruxolitinib, and the contribution of prior high-dose corticosteroid therapy to the improvement in COP and respiratory status cannot be excluded. Therefore, the improvement in ALS should not be attributed solely to ruxolitinib. Rather, ruxolitinib may have helped control steroid-dependent COP and enabled gradual PSL reduction without relapse, thereby minimizing steroid-induced impairment of alveolar wound healing and potentially contributing to the improvement in PM.

The reported risk factors include age > 38 years, male sex, multiple transplants, a history of grade 3-4 acute GVHD, extensive chronic GVHD, COP, and a history of invasive fungal infection [1,2]. In this case report, the patient was a 60-year-old man with extensive chronic GVHD and COP as risk factors. Acute and chronic GVHD are occasionally observed after allogeneic HSCT, and COP is occasionally observed with GVHD [3,6-9]. The incidence of COP was reported to be 2.0% in Japan, and systemic steroid therapy as first-line treatment has shown good responses [9]. Some patients with COP become steroid-refractory or relapse during steroid tapering [8]. In this case, the patient experienced a relapse of COP immediately after the discontinuation of PSL. Due to severe respiratory failure, BAL could not be performed; however, infectious etiologies were considered unlikely based on negative results for nasopharyngeal respiratory virus panel PCR and serum galactomannan antigen, and the patient was diagnosed with steroid-dependent COP.

KL-6 is a mucin-like high-molecular-weight glycoprotein expressed in type II pneumocytes, and increased KL-6 levels reflect type II alveolar epithelial cell damage and remodeling [14]. Serum KL-6 levels are elevated in interstitial lung disease, including COP, and their elevated levels can indicate disease activity or progression [14,15]. Pulmonary fibrosis and high serum KL-6 levels suggest persistent COP activity [16]. In the present case, the patient with COP had elevated serum KL-6 levels at the time of COP relapse, and his respiratory condition remained unstable despite high-dose corticosteroid therapy before the development of ALS. After the initiation of ruxolitinib treatment, serum KL-6 levels decreased in parallel with radiographic improvement of PM and improvement in oxygenation. Although serum KL-6 is primarily a marker of interstitial lung disease activity, including COP, the parallel improvement in KL-6 levels and PM following ruxolitinib treatment may suggest that serum KL-6 reflects the overall disease activity in patients with COP-associated ALS.

In the REACH3 study, common hematological adverse events associated with ruxolitinib included anemia, thrombocytopenia, and neutropenia, with reported incidence rates of 29.1%, 21.2%, and 10.9%, respectively. Laboratory abnormalities such as increased alanine/aspartate aminotransferase and increased creatinine have been reported as adverse events associated with ruxolitinib [7]. In this case, the adverse events associated with ruxolitinib included anemia (CTCAE grade 2), thrombocytopenia (CTCAE grade 4), neutropenia (CTCAE grade 2), and increased alanine aminotransferase level (CTCAE grade 2), which were consistent with those reported previously [7,17]. The patient died of acute respiratory failure after transfusion therapy on day 392 after the PBSCT. TRALI was clinically suspected; however, transfusion-associated circulatory overload and cardiogenic pulmonary edema could not be completely excluded. Although a causal relationship between ruxolitinib-induced thrombocytopenia and the need for transfusion could not be excluded, this fatal event was not considered a direct complication of ruxolitinib. Therefore, patients undergoing HSCT should be monitored for pancytopenia, hepatotoxicity, and infection during ruxolitinib treatment.

## Conclusions

This case demonstrated improvement in PM after the initiation of ruxolitinib treatment. Ruxolitinib was associated with improved oxygenation, radiographic improvement, and successful tapering and discontinuation of corticosteroids without relapse of organizing pneumonia. However, because this is a single-patient case report without a comparator, a causal relationship between ruxolitinib and the resolution of ALS cannot be established. In addition, spontaneous improvement of PM cannot be excluded. Therefore, ruxolitinib may have helped control steroid-dependent organizing pneumonia and facilitated corticosteroid tapering, with associated improvement in PM. Further prospective studies are needed to clarify the efficacy and safety of ruxolitinib for post-transplant ALS.
